# Neutrophil Extracellular Traps in Digestive Cancers: Warrior or Accomplice

**DOI:** 10.3389/fonc.2021.766636

**Published:** 2021-11-19

**Authors:** Yuxin Chen, Lulu Han, Xiaoyan Qiu, Gang Wang, Junnian Zheng

**Affiliations:** ^1^ Cancer Institute, Xuzhou Medical University, Xuzhou, China; ^2^ Jiangsu Center for the Collaboration and Innovation of Cancer Biotherapy, Cancer Institute, Xuzhou Medical University, Xuzhou, China; ^3^ Center of Clinical Oncology, The Affiliated Hospital of Xuzhou Medical University, Xuzhou, China

**Keywords:** neutrophil extracellular trap, cancer, digestive system, immunity, targeted therapy

## Abstract

Characterized as a complex of extracellular DNA fibers and granule proteins, neutrophil extracellular traps (NETs) are generated specifically by neutrophils which play a critical role in host defense and immune regulation. NETs have been initially found crucial for neutrophil anti-microbial function. Recent studies suggest that NETs are involved in tumorigenesis and cancer progression. However, the function of NETs in cancer remains unclear, which might be due to the variation of research models and the heterogeneity of cancers. Although most of malignant tumors have similar biological behaviors, significant differences indeed exist in various systems. Malignant tumors of the digestive system cause the most incidence and mortality of cancer worldwide. In this review, we would focus on research developments on NETs in digestive cancers to provide insights on their role in digestive cancer progression and future research directions.

## Introduction

As a subset of innate immune cells, neutrophils play a critical role in host defense against microbial infection, and are traditionally recognized as a short-life and terminally differentiated cell (1). Thus, previous studies on neutrophil function on the tumor are limited. However, an increasing number of studies have shown the association of neutrophils with cancer progression, although the roles of neutrophils in cancer progression remain a controversial issue, which might be due to the spatial-temporal tumor microenvironment (2). One of the ways neutrophils participate in cancer progression is through NETosis, wherein neutrophils expel their genomic DNA decorated with proteins (3).

Neutrophil extracellular traps (NETs) are the products of NETosis and were first described by Brinkmann et al. (4) as an extracellular fiber formed by granule proteins and chromatin of neutrophils to capture and kill invading bacteria; NETs were later found to be involved in host immune defense against fungi (5), parasites (6) and viruses (7). However, dysregulated NETs cause the pathogenesis of immune-related diseases. Recently, NETs have been reported to be associated with the immunothrombosis and disease severity caused by COVID-19 (8, 9) and considered as a prognostic marker (10) and therapeutic target (11). Studies have shown that NETs play a distinct role in cancer. On the one hand, NETs exert their anti-tumor function by directly killing cancer cells or indirectly cooperating with other immune cells. On the other hand, NETs have been found to contribute to cancer progression in several ways, such as inhibiting apoptosis and inducing tumor angiogenesis (12). Although numerous studies have shown that NETs may be more inclined to promote tumor progression, the actual role of NETs in cancer remains unknown, mainly due to the diversity of NET-related components, the complexity of the tumor immune microenvironment, and the differences of various tissues and organs.

The digestive system is composed of the oral cavity, esophagus, stomach, small intestine, colorectum, liver, and pancreas; the malignancy of these components has severely damaged public health (13). According to the global cancer statistics for 2018, the incidence and mortality of digestive cancers approximately account for 29% and 30% in all types of cancers worldwide, respectively; these statistics indicate that digestive cancers are not only the most common cancer but also the most frequent cause of cancer-related death all over the world. Among the digestive cancers, colon cancer and gastric cancer represent the top incidence and mortality, respectively (14). Neutrophils have been demonstrated to play a vital role in digestive cancer progression (15). Neutrophil-to-lymphocyte ratio (NLR) is negatively associated with the prognosis of digestive cancers, such as oral squamous cell carcinoma (OSCC) (16), esophageal cancer (17), and gastrointestinal malignancies (18). NETs, acting as a weapon of neutrophils, have also been found to be involved in the progression of digestive cancers (19).

In this review, the formation and degradation of NETs are concisely described first. Second, the relationship between NETs and cancer is briefly summarized from the aspects of tumor growth, metastasis, angiogenesis, and thrombus. the effect of NETs on digestive cancers is analyzed in the third part. Lastly, the possible targeting strategies and future research directions on NETs for cancer therapy are discussed and predicted.

## Formation and Degradation of NETs

The process of NET formation is defined as NETosis. Upon stimulation, two different ways for NETosis have been proposed, namely, suicidal NETosis and vital NETosis (non-lytic NETosis) (20). In the suicidal approach, decondensed chromatin in active neutrophil is expelled *via* nuclear envelope and plasma membrane rupture followed by neutrophil death (21), whereas non-lytic NETosis is achieved through the rapid release of NETs components and the reservation of the neutrophil’s phagocytic function (20–22). Most of the existing studies have focused on the understanding and effect of NETosis compared with the non-lytic NETosis, which helps in illustrating the nature of NETs.

Regardless of how NETs are formed, the critical process is chromatin decondensation, which is mainly triggered by reactive oxygen species (ROS) and peptidylarginine deiminase 4 (PAD4). Induced by stimulus, such as phorbol myristate acetate and lipopolysaccharide (LPS) (23), nicotinamide adenine dinucleotide phosphate (NADPH)-mediated ROS production leads to the activation and nucleus translocation of myeloperoxidase (MPO) and neutrophil elastase (NE) (24), which subsequently promotes the degradation of specific histones and chromatin decondensation. Moreover, other stimuli, including immune complexes, ionomycin, and nicotine, could induce formation of NETs *via* mitochondrial ROS independently of NADPH oxidase (25–27). Although these reports suggest that ROS plays a crucial role in the release of NETs, NET formation can transpire without ROS production. Non-lytic NETosis has been reported to be independent from ROS production (22).

Another nuclear chromatin protein, DEK, has been reported to be involved in NETosis. NETosis is restricted in DEK-deficient neutrophils and can be subsequently rescued by recombinant DEK protein; this finding suggests that DEK is crucial in NETs formation (28). Chromatin depolymerization, another critical step for NET formation, depends on histone deamination or citrullination, which is driven by the ribozyme PAD4 (29, 30). A recent study shows that PAD4 inhibitors prevent NET formation in sepsis and tumor models (31, 32), and PAD4-deficient neutrophils lose the ability to release NETs after stimulation with LPS and tumor necrosis factor (33).

The mechanism of NETs degradation requires further exploration. Studies have shown that during infection, NETs can last for several days (34) and can be gradually degraded and cleared by the nuclease DNase I (35). DNase I injection can rapidly degrade NET-related DNA, while NET-related proteins still exist after DNA degradation (31). DNase I has been found to promote the phagocytosis of NETs by macrophages *in vitro*, suggesting that the phagocytosis of macrophages may be one of the mechanisms for clearing NETs (36).

Notably, the specific characteristics of NETs, such as mechanisms of formation and degradation, and their exact components, have not been fully elucidated, hampering NETs evaluation in the laboratory setting. NETs can be considered as a type of DNA-protein complex, therefore, several markers have been used to detect the level of NETs at the laboratory, such as cell-free DNA (cfDNA), MPO, NE, citrullinated histone H3 (citH3), citH3-DNA and MPO-DNA (10, 27, 37, 38). For example, the levels of cfDNA and MPO-DNA are usually employed to measure the NET formation in the serum, plasma or neutrophil culture medium. The protein components of NETs, including MPO, NE, and CitH3, can also be used to determine NET formation in tissue samples by immunohistochemistry and immunofluorescence. However, the golden standard to detect NETs has not yet been illustrated. Therefore, the use of combined markers to evaluate NET formation is reasonable.

## Factors of Tumor-Induced NET Formation

NETs have been found in the primary tumor, metastasis, or circulating system in the context of malignant tumors (37, 39, 40). The effect of NETs in cancer was first presented in 2012 (41), and the finding on NETs in cancer tissue was demonstrated in 2013 (42). Several factors, such as granulocyte colony-stimulating factor (G-CSF) (43) and interleukin 8 (IL-8) (44), were also found to stimulate NETs formation in cancer. However, the mechanisms of tumor-induced formation of NETs remain unclear.

Three major aspects may affect NET formation in the presence of cancers. One is the tumor cell itself, which may be the primary factor to affect NETs formation. IL-8, also known as CXCL8, is a major chemokine that promotes the infiltration of polymorphonuclear leukocytes into tissue (45); IL-8 has also been found to be involved in cancer progression (46). Numerous studies confirm that neutrophils are prone to release NETs under the induction of IL-8 in solid and lymphoid-hematopoietic system tumors (44, 47–54). G-CSF has been used to increase circulating neutrophils to prevent neutropenia-associated serious infections after systematic chemotherapy (55). However, reports have shown that endogenous G-CSF promotes the progress of several types of cancers, such as neuroblastoma and breast cancer (56, 57), and tumor-derived G-CSF plays a critical role in NET formation in breast cancer and lung cancer (39, 41, 58). Tumor cells can also induce NET formation by secreting a variety of other stimulating factors, such as exosome, transforming growth factor-β (TGF-β), interleukin-1β (IL-1β), extracellular RNA (exRNA), mitochondrial DNA (mtDNA), high mobility group box-1 (HMGB1), cathepsin C (CTSC), tissue inhibitor of metalloproteinases-1 (TIMP-1), CXCL5 (59–69).

Stromal cells in the tumor microenvironment (TME), such as immune cells, endothelial cells, fibroblasts, and adipocytes, also contribute to NET formation (70, 71). Murine lung epithelial-12 cells, activated by Lewis lung cancer cells, provoke NETs release by producing IL-1β (63). IL-17, a cytokine secreted by TH17 cells, has been found to induce NET formation in pancreatic ductal adenocarcinoma (PDAC) (72). Inorganic polyphosphate, a natural molecule, also has been recently found to be a stimulus for NETs formation (73). Mast cells in colon cancer, instead of non-malignant conditions, have been found to generate inorganic polyphosphate, which may promote NETs formation in colon cancer (74). Numerous studies have been conducted on the role of platelets in NET formation, considering that platelets are closely related to the development and progression of cancer (75). Research has shown that activated platelets in pancreatic cancer promote tumor-associated neutrophils to form NETs (76). Another study further demonstrated that activated platelets induce NETs formation *via* secreting HMGB1 (77). Moreover, HMGB1 is identified as one of the components in NETs (78), which suggests that HMGB1 released by neutrophils may encourage other neutrophils to form NETs. In pancreatic cancer and melanoma, amyloid β, derived from cancer-associated fibroblasts, facilitates NETs formation *via* CD11b in a ROS-dependent manner (79). Aside from the abovementioned stimuli, complement 3a (C3a), a type of complement, has also been found to promote the formation of NETs (80).

The third type of factor that induces NET formation is metabolic factors. Plasma redox imbalance is found to encourage NET formation in lung cancer (81). Free fatty acid was discovered to be a stimulus of NET formation in nonalcoholic steatohepatitis, and NETs promote the occurrence and development of hepatocellular carcinoma (82). Although studies have shown that tumors can induce neutrophils to excrete DNA and protein complexes to form NETs, only a few studies have explored the factors that induce NET formation in tumors. Whether other cells or immune components are involved in the regulation of NETs in TME requires further research. In particular, only a few studies have explored the mechanisms by which these stimulus factors lead to increased formation of NETs. Thus, future studies may need to focus on the underlying mechanisms of induction factors. **Table 1** collected specific information about inducer factors that promote NET formation in tumors.

**Table 1:** Detailed information about NETs induced by tumors.

Stimuli	Receptors	Intermediators	Summary of findings	Diseases	Refs
CXCR1/CXCR2 ligands	CXCR1/2	–	Tumor-induced NETosis is mainly triggered by CXCR1 and CXCR2 ligands, which could be constrained by CXCR1 and CXCR2 inhibitors.	Melanoma, colon carcinoma and breast cancer	(47)
IL-8	CXCR2	Src, p38 and ERK	NETosis is induced by IL-8 *via* IL-8-CXCR2, which could be inhibited by IL-8 antibody, CXCR2 inhibitor, and pharmacological inhibition of Src, p38 and ERK respectively.	DLBCL	(48)
	CXCR2	–	Plasmatic IL-8 plays a crucial role in priming neutrophils for NETs formation in patients.	CLL	(49)
	CXCR2	PI3K-AKT-ROS	IL-8 secreted by tumor cells recruits neutrophils to tumor site and promotes NETs formation.	Glioblastoma	(50)
	CXCR2	–	IL-8 derived from patients’ plasma and culture medium of HT29 cell could provoke neutrophils to extrude NETs.	Colon carcinoma, NSCLC and prostate cancer	(44)
	–	–	There is a positive feedback between tumorous IL-8 and NETs to facilitate CRC liver metastasis.	CRC	(51)
	–	–	Breast cancer cells promote the NETs formation *via* secreting IL-8.	Breast cancer	(52)
	–	–	Tumor cell IL-8 simulates the formation of NETs to accelerating thromboembolism.	Gallbladder cancer	(53)
	–	NADPH	HCC cells-derived IL-8 triggers NETs formation depended on NADPH oxidase, which could be inhibited by IL-8 antibody and NOX2 inhibitor apocynin respectively.	HCC	(54)
G-CSF	–	–	G-CSF released from LLC cells primes intertumoral neutrophils toward undergo NETosis process, and neutrophils of peripheral blood isolated from LLC-bearing mice have a high tendency to form NETs.	Lung cancer	(58)
	–	–	G-CSF potentiates the ability of neutrophils to form NETs upon platelet-activating factor, which could be significantly diminished by G-CSF neutralizing antibody treatment.	Breast cancer	(41)
	–	–	G-CSF secreted by 4T1 cells promotes neutrophils to form NETs, and human recombinant G-CSF also can induce the formation of NETs on human neutrophils.	Breast cancer	(39)
Exosomes	–	IL-8	The formation of NETs can be induced by mutant KRAS exosomes through the upregulation of IL-8.	CRC	(59)
	–	–	Exosomes derived from 4T1 cells can promote G-CSF primed neutrophils to form NETs.	Breast cancer	(60)
TGF-β	TGF-βR	PI3K	rhTGF-β can lead to the increased formation of NETs.	Precancerous lesion of OSCC	(61)
IL-1β	IL-1βR	G-CSF	IL-1β stimulates the production of NETs through upregulating the level of circulating G-CSF.	Breast cancer	(62)
	–	–	IL-1β secreted by epithelial cells provokes NETs, which can be aborted by IL-1β inhibitor.	Lung cancer	(63)
ex-RNA	–	–	ex-RNA indirectly promotes the NETs formation by stimulating epithelial cell to secrete IL-1β.	Lung cancer	(63)
mtDNA	–	–	mtDNA renders NETosis through NOX2-independent pathway.	Ovarian cancer	(64)
IL-17	IL-17R	–	IL-17 secreted by TH17 cells contributes to NETs formation.	Pancreatic cancer	(70)
Polyphosphate	–	–	Polyphosphate expressed by mast cells may prime neutrophils to extrude NETs.	Colon cancer	(74)
Activated platelet	–	–	Platelets activated by Aspc-1 cells stimulate NETs release.	Pancreatic cancer	(76)
HMGB1	TLR4	MAPK-ERK	HMGB1 released from LLC cells promotes the NETs formation through TLR4-MAPK pathway.	Lung cancer	(65)
	TLR4	–	HMGB1, derived from urothelial cancer cell line (UM-UC3) promotes NET formation through a TLR4-dependent manner.	Bladder cancer	(68)
	–	–	Platelet-derived HMGB1 induces NETosis, which could be markedly obstructed by anti-HMGB1 antibody.	CRC	(77)
CXCL5	–	–	CXCL5, derived from pancreatic cancer cells, primes tumor associated neutrophil to form NETs.	Pancreatic cancer	(69)
CTSC	Proteinase 3	IL-1β	CTSC, associated with 4T1 cells potentiates the NETs formation by proteinase 3-IL-1β pathway.	Breast cancer	(66)
TIMP1	CD63	ERK	Pancreatic cancer TIMP1 stimulates neutrophils to release NETs *via* activating CD63-ERK pathway.	Pancreatic cancer	(67)
Amyloid β	CD11b	ROS	Amyloid β promotes the NETs formation in a ROS-dependent mechanism.	Pancreatic cancer and melanoma	(79)
C3a	C3aR	–	C3a promotes LDN to release NETs through C3aR-dependent pathway.	Small intestinal cancer	(80)
Plasma redox imbalance	–	–	Plasma redox imbalance caused by albumin oxidation triggers NETosis by an inflammation way.	HNSCC	(81)
Free fatty acid	–	–	Elevated free fatty acid stimulates NETs formation.	HCC	(82)

CLL, Chronic lymphocytic; CLL, Chronic lymphocytic leukemia; DLBCL, Diffuse large B-cell lymphoma; HNSCC, Head and neck squamous cell carcinoma; HCC, Hepatocellular carcinoma; NSCLC, Non-small cell lung cancer OSCC; Oral squamous cell carcinoma; HNSCC, Head and neck squamous cell carcinoma; CTSC, Cathepsin C; C3a, complement 3a; C3aR, complement 3a receptor; exRNA, extracellular RNA; HMGB1, High mobility group box-1; mtDNA, mitochondrial DNA; NADPH, Nicotinamide adenine dinucleotide phosphate; NOX2, NADPH oxidase 2; TIMP-1, Tissue inhibitor of metalloproteinases-1. No determined receptors or intermediators lead to the appearance of short bars.

## Function of NETs on Cancer Progression

Studies on the role of NETs in tumors are still limited. Acting as an arm of neutrophils, NETs have been demonstrated to perform an anti-tumor or a pro-tumor function on cancer progression. NETs induced indirectly by retinoic acid exert an anti-tumor function by enhancing the cytotoxicity of neutrophils to breast cancer (83), and Bacillus Calmette-Guerin-NETs inhibit the proliferation and metastasis of bladder cancer (84). NETs can also induce the apoptosis of colon cancer cells to suppress cell proliferation *in vitro* (85). CD16^high^ CD62L^dim^ neutrophils, a subset of neutrophils, is characterized by high expression of CD11b and CD18; NETs derived from these neutrophils have been demonstrated to play an anti-tumor role in head and neck squamous cell carcinoma (86). Recent studies have shown that NETs are correlated with better outcome of ovarian cancer patients (87) and may play an antineoplastic role by inducing the necrosis of melanoma cells (88). As compared with limited anti-tumor reports, several studies provide contrasting results suggesting that NETs mainly play a role in promoting tumor, which is supported by clinical data (89–91).

NETs have been demonstrated to be involved in the entire process of tumor progression, including tumor cell proliferation, adhesion, migration, and metastasis in several *in vitro* and *in vivo* models. Moreover, NETs have also been demonstrated to suppress the anti-tumor immune response and to promote cancer-associated thrombosis formation. In addition to directly affecting tumor cells, NETs also contribute to tumor-associated kidney injury in mice (92) and are related to cancer-associated stroke and disseminated intravascular coagulation (93, 94). These cancer-related vascular and organ complications may be partly due to vascular dysfunction induced by NETs. Endothelial cells are forced to undergo morphological changes under the influence of NETs, including retraction from cell-cell junctions and a more pro-coagulative phenotype (95). The vascular functions of organs have also been found to be affected by NETs instead of tumor cells themselves (43, 96). However, only a few studies have been conducted on the specific mechanism by which NETs contribute to tumor progression. Next, we will focus on the mechanism by which NETs affect tumor progression.

Given the complexity of NET components, several studies have identified NETs in their entirety to analyze their role in tumors. Researchers found that NETs promote the migration and invasion of cancer cells by activating AKT and STAT3 pathways (52). NETs also inhibit apoptosis by regulating NF-κB p65, BCL-2, and Bax expression (97). NETs have been found to be involved in the progression of diffuse large B-cell lymphoma (DLBCL) and colorectal cancer *via* TLR9-MAPK pathway (48, 98). Furthermore, a tumoral inflammatory response could be triggered by NETs through the activation of the TLR4/9-COX2 axis to fuel metastasis (38). However, considering the complexity of NETs, it is difficult to accurately clarify the role of NETs in tumors from a holistic perspective. Therefore, the roles of NET-related components have received more attention in recent years. DNA expelled by activated neutrophils serves as a cornerstone of NETs structure; and has been discovered to play a critical role in the function of NETs, because several kinds of studies demonstrate that DNase I could effectively inhibit the pro-tumor function of NETs by degrading extracellular DNA (37, 89). NET-DNA could act as a chemotactic factor to facilitate the migration of tumor cells to the metastatic site (99). In this process, NET-DNA could interact with extracellular N terminus of the coiled-coil domain containing protein 25 (CCDC25), a transmembrane protein expressed on tumor cells; C terminus of CCDC25 then recruits integrin-linked kinase (ILK), which subsequently stimulates β-parvin-RAC1-CDC42 cascade to facilitate tumor progression (99). NET-DNA could also promote the aggregation of platelets by binding to the receptor for advanced glycation end products (RAGE) of platelets, which contributes to the hypercoagulable state of pancreatic adenocarcinoma (100). NET-DNA could also activate the pancreatic stellate cell by upregulating the expression of p65 and pERK (101).

Apart from DNA, the complexity of NETs is determined by its protein content. Studies have confirmed that NETs include more than 20 different kinds of proteins, such as NE, MPO, calprotectin, cathelicidins, defensins, and HMGB1 (50, 102). Among these proteins, only a few studies have explored the effects and specific mechanisms of NET-related protein components on tumor progression. NET-released NE is capable of not only inducing mitochondrial dynamics, but also promoting MC38 cell proliferation by stimulating TLR4 signaling which then activates the p38-PCG1α pathway (103). Tumor recurrence after treatment remains one of the main causes of cancer-associated death. Usually, metastatic cancer cells may undergo a long period of dormancy before the formation of detectable metastasis. When encountering appropriate or specific stimuli, dormant tumor cells re-enter the proliferation state and cause tumor recurrence (104). NETs have been found to mediate this process. During this process, laminin protein in the extracellular matrix (ECM) could be cleaved by NE and matrix metalloproteinase 9 (MMP9) coating on NETs; the cleaved subset of laminin could interact with integrin α3β1 of tumor cells, which then activates the focal adhesion kinase (FAK), MAP kinase ERK kinase (MEK), and myosin light chain 2 (MLC2) to finally awaken the dormant cancer cells (105). HMGB1 is another protein of NETs-related components under investigation. NET-HMGB1 interacts with the TLR9 of tumor cells and then stimulates TLR9 signaling which subsequently activates p38 and JNK pathways for tumor proliferation, adhesion, migration, and invasion (89). In a murine model of glioblastoma, HMGB1 contributes to IL-8 production from tumor cells through HMGB1-RAGE-ERK1/2-NF-κB pathway, which may further attract circulating neutrophils to the tumor site and stimulate them to form NETs (50). Histone is also a critical protein component of NETs, and has been considered a factor that induces angiogenesis (106). Furthermore, NET-associated IL-1β is involved in the epithelial-to-mesenchymal transition (EMT) process of pancreatic cancer by activating EGFR-ERK pathway (107). NET-fibronectin mediates the adhesion of several human cancer cells by interacting with integrin family, such as αvβ5, αvβ3, and α5β1 (108). NET-integrin β1 promotes the interaction between NETs and A549 cells, leading to the adhesion of lung cancer cells to liver sinusoids (109).

**Figure 1:**
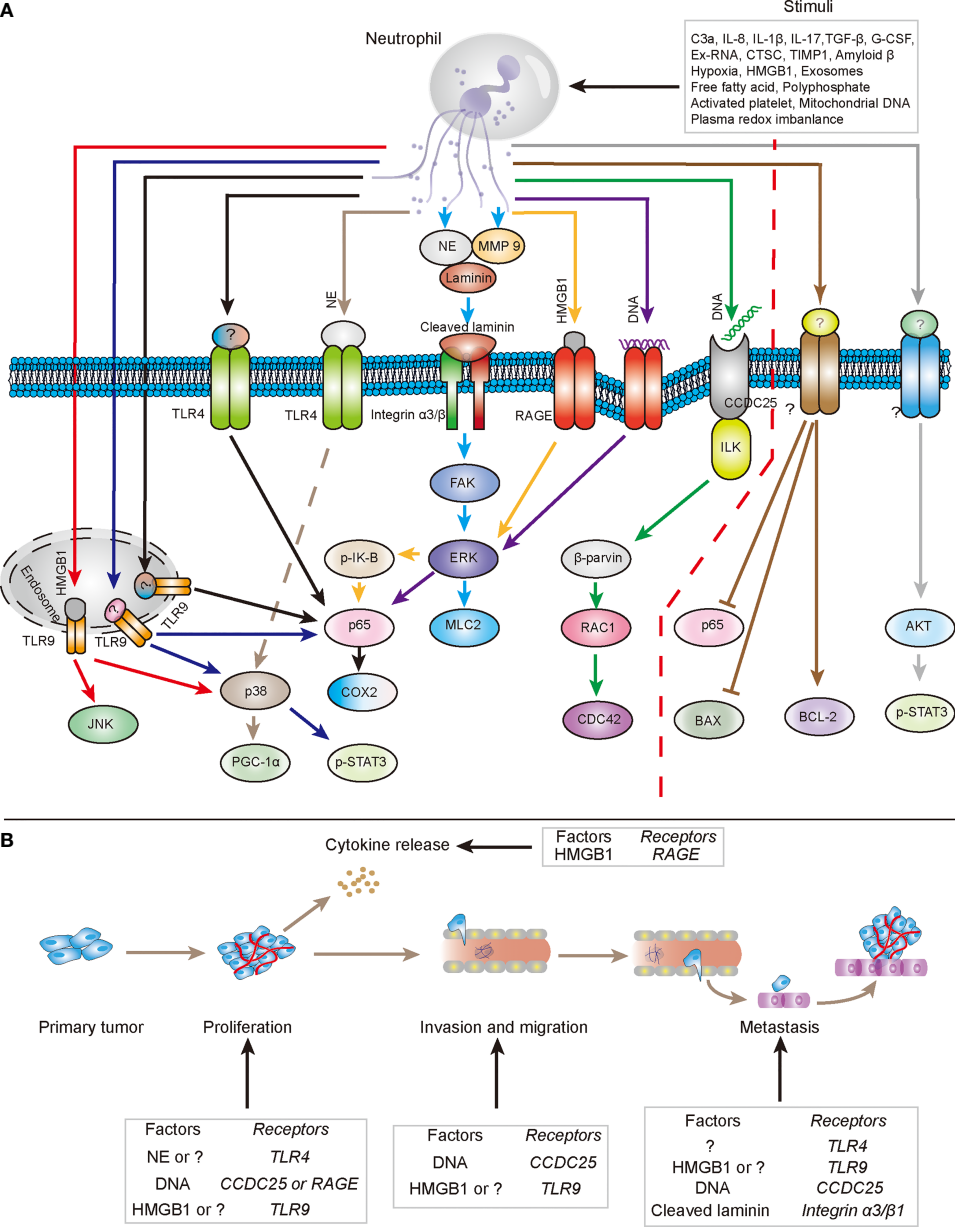
The mechanisms by which NETs directly promote tumor progression. **(A)** In several types of stimuli in tumor tissues, neutrophils expel their DNA and cytoplasmic proteins to form extracellular structure to participate in tumor progression. NETs-DNA could interact with RAGE and CCDC25 respectively to be involved in the transduction of intracellular tumor-promoting signals, which facilitate tumor progression. Additionally, NETs associated protein components, such as NE, MMP9 and HMGB1, also could cooperate with respective receptor to stimulate downstream pathways to fuel the malignancy of tumors. Question marks (?) represent relevant functional components of NETs and related receptors that have not been clearly stated (especially the right side of red dotted line). **(B)** Components of NETs involved in the cytokine release, proliferation, invasion, migration and metastasis of tumor cells through respective relevant receptors.

Part of the mechanism by which NETs promote tumor progression has been revealed in **Figure 1**. TLRs, integrin family, RAGE, and CCDC25 have been proven to be involved in signal transduction by direct NET binding. Furthermore, activation of NF-κB and MAPK plays an important role in downstream signal transduction. However, numerous questions still need to be answered. In particular, the underlying mechanism of other NET-related proteins in tumorigenesis and development remains poorly understood. Consequently, future studies may need to further explore the components of NET-related proteins, the effect and mechanism of various protein components on tumors, and the characteristics of NETs performing anti-tumor effects.

## Role of NETs in Digestive System Cancers

**Figure 2:**
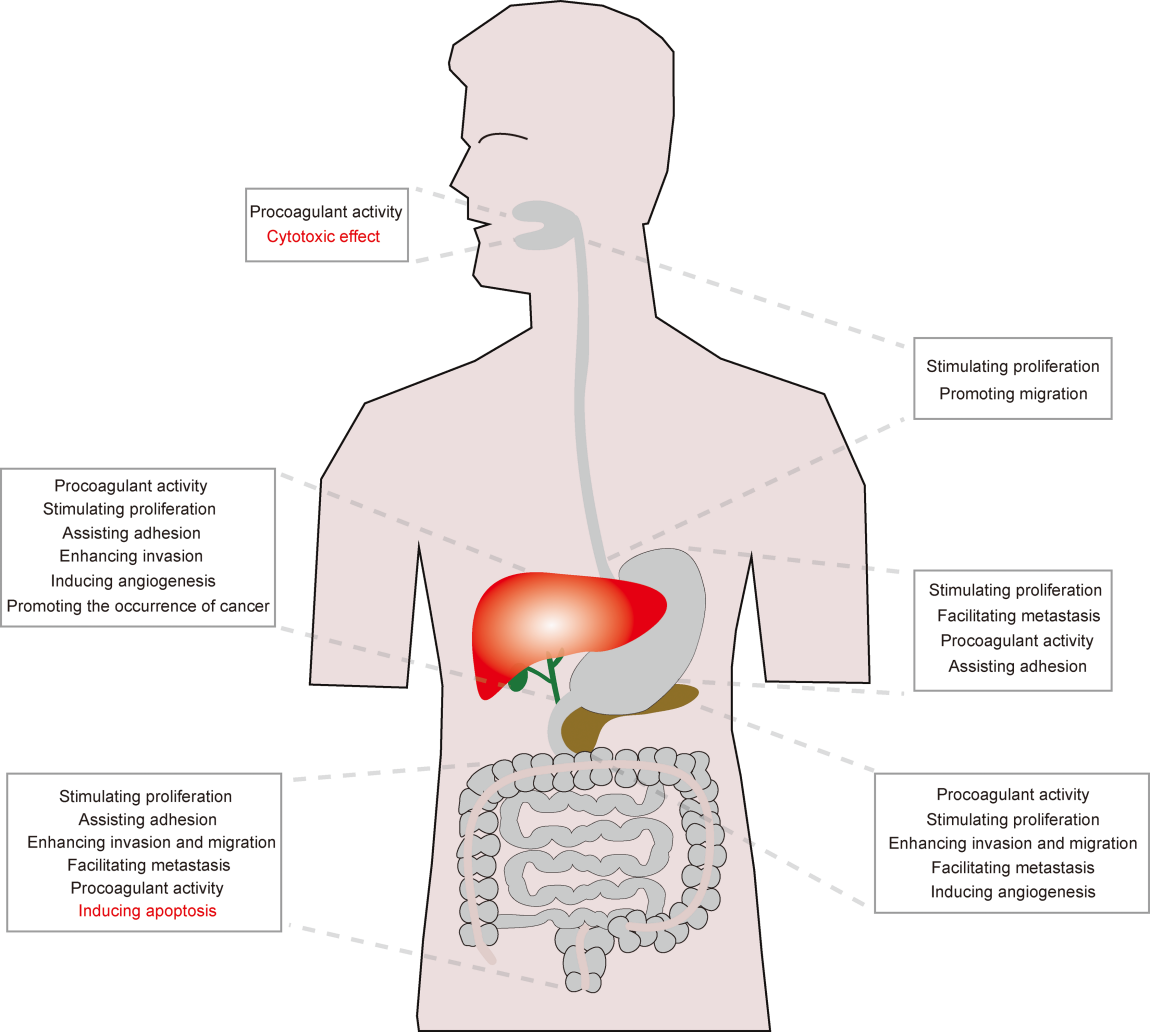
The main effect of NETs on digestive cancers. Although the effect of NETs on OSCC and colon carcinoma remains unclear, NETs mainly play a pro-tumor role in esophageal, gastric, pancreatic, liver and biliary cancers.

The function of NETs on digestive system cancers has attracted widespread attention in recent years. Several reports have shown that NETs may perform an anti-tumor effect, but majority of existing studies suggest that NETs can exacerbate tumor-related symptoms or accentuate tumor growth, which affect the survival of patients with cancers, especially pancreatic cancer and HCC. The main functions of NETs on digestive system cancers are presented in **Figure 2**.

### Oral Cavity Cancer

OSCC is the most common tumor of oral malignancies. Neutrophils have been found to be involved in oral cancer progression (110, 111), and inhibiting tumor-associated neutrophils could repress OSCC metastasis (112). Meanwhile, peripheral NLR has been demonstrated to be an independent predictor for the evaluation of overall survival (OS) for OSCC patients (113). However, only a few studies have focused on the role of NETs in oral cancers.

An increase of NET formation has been discovered in the coculture system of neutrophils with OSCC cells (114). In addition, a study based on oral precancerous lesions (oral lichen planus) suggests that neutrophils stimulated by TGF-β release more NETs, which may indicate that “N2” neutrophils may be more inclined to form NETs by upregulating PI3K after stimulation. Thus, the increased release of NETs may also be one of the mechanisms by which “N2” neutrophils promote tumor progression (61). Moreover, peripheral neutrophils in patients with Stage III/IV OSCC produce more NETs compared with healthy controls or Stage I/II patients; NETs were also found to promote the transformation of human vascular endothelial cells to a procoagulant phenotype, suggesting that elevated NETs are positively correlated with disease severity and coagulation disorders (115). However, Marzena and colleagues found that NETs in patients with inflammation and with Stage III/IV OSCC are markedly higher than those found in Stage I/II OSCC patients and healthy donors; these findings may indicate that NETs participate in the transformation of epithelial cells to malignant cells due to the stimulation of inflammation, and that NETs may play a distinct role at different stages of OSCC (116). Human neutrophil peptide-1 (HNP1) has been demonstrated to be a NET-related component (117), and found to inhibit the proliferation of OSCC cells *in vitro* (118), indicating that NET-related HNP1 contributes to the suppression of tumor progression.

Taken together, in response to the varying findings presented earlier, the effect and related mechanisms of OSCC on NET formation and the function of NETs on OSCC remain unclear and require further study.

### Esophageal Cancer

Esophageal cancer is one of the most common tumors of the digestive system, which can be roughly divided into squamous cell carcinoma and adenocarcinoma. Regardless of the pathological type, most patients present in an advanced stage at the time of diagnosis and 80% of patients live no more than five years (119). Therefore, exploring the mechanism of esophageal cancer progression is the top priority for current research.

Circulating NETs in patients with advanced esophagogastric adenocarcinoma are significantly higher as compared with those in Stage I/II patients and healthy individuals; these findings suggest that NETs may be related to the progression of esophagogastric carcinoma (37). Another research also found that circulating cfDNA levels in patients with esophageal squamous cell carcinoma (ESCC) are significantly higher than those in control patients with benign diseases, and serum cfDNA levels are closely associated with tumor size and the host’s inflammation status, such as the number of neutrophils in the peripheral blood of patients (120). Recently, a retrospective study demonstrated that tumor-infiltrating NETs are associated with dismal OS and disease-free survival (DFS) (121). NE, derived from activated neutrophils, could also promote the proliferation and progression of esophageal cancer cells by stimulating the release of cytokines, such as TGF-β, vascular endothelial growth factor (VEGF), and platelet-derived growth factor-AA (PDGF-AA), which could be blocked by NE inhibitor (sivelestat) (122). However, studies on the reasons for increased NET levels in patients with esophageal cancer are still limited. A study has shown that IL-8 levels tend to be higher in patients with esophageal cancer as compared with those of healthy donors, and that IL-8 levels are closely associated with tumor size and lymph node metastasis, suggesting that increased IL-8 may be related to the progression of esophageal cancer (123). Considering that neutrophils are inclined to form NETs upon IL-8 stimulation (124), NET formation is believed to be correlated with IL-8 in esophageal cancer, which needs further confirmation in future studies.

Tumor progression is usually accompanied by a hypercoagulable state of blood which is closely related to the patient’s prognosis. Fibrinogen and NLR score have been found to be related to tumor size, stage, invasion, and lymph node metastasis of esophageal cancer, indicating that the hypercoagulable state of blood may be closely correlated with the progression of esophageal cancer (125). However, whether NETs are involved in the formation of blood hypercoagulability in esophageal cancer remains unclear, and thus require further study.

Intriguingly, several studies aimed to explore whether sivelestat sodium, a type of NE inhibitor, could attenuate the postoperative complications of esophageal cancer patients; and these studies found that sivelestat sodium could significantly lighten postoperative hypoxia, partially reduce systemic inflammation, and maintain postoperative circulatory status, which is beneficial for the recovery of patients (126–128). However, the effect of NE inhibitor treatment on the subsequent progression of esophageal cancer is difficult to ascertain without the publication of follow-up date. Notably, research on NETs focuses on ESCC, and studies on esophageal adenocarcinoma are limited. Barrett’s esophagus is considered a precancerous lesion of esophageal adenocarcinoma. Studies have shown that increased ROS levels of neutrophils significantly lead to esophageal mucosal injury in Barrett’s esophagus, which could be retarded by the MPO inhibitor azide (129). this finding suggests that the ROS of neutrophils may be involved in the malignant transformation of Barrett’s esophagus. Considering that ROS is one of the key factors in the NET formation, whether NETs are connected with the malignant transformation of Barrett’s esophagus is worthy for further investigation.

### Gastric Cancer

Although surgical treatment is still the main therapy for patients with gastric cancer, surgery-related complications, recurrence, and distant metastasis are considered the main cause of death. Rihito and colleagues found that low-density neutrophils (LDN), derived from abdominal cavity lavages after abdominal surgery, are prone to NET formation (130). NETs could trap the human gastric cancer cell lines (i.e., MKN45, OCUM-1, NUGC-4) *in vitro* to promote their proliferation (130), facilitate a more aggressive mesenchymal phenotype of AGS cells (131), and strongly augment the metastasis of MKN45 cells on peritoneum, which could be inhibited by IP administration with DNase I (132); these findings suggest that NETs play an important role in peritoneal metastasis of gastric cancer. In view of NETs that can trap tumor cells and promote their survival, whether NETs in the circulatory system can trap gastric cancer cells and assist them in the metastasis to a distant organ are yet to be determined.

Circulating neutrophils in gastric cancer patients are prone to form NETs spontaneously compared with those in healthy donors (133), NET levels are significantly associated with neutrophil count and NLR in the peripheral blood (134). Roni and colleagues found that circulating NETs are significantly elevated in patients with advanced gastric tumors, compared with those in local cancer patients and healthy controls, and closely associated with disease stage (37). Interestingly, NETs could serve as a prognostic factor, which may be more sensitive to predicting the outcome of patients compared to carcinoembryonic antigen (CEA) and carbohydrate antigen 199 (CA199) of serum (134). Moreover, increased LDN production has been found to be positively correlated with operative time and intraoperative blood loss, and LDN is prone to form NETs which served as a “carrier” and an “armor” for transporting and protecting gastric cancer cells to facilitate distant metastasis (135). However, surgery stress is not a necessary condition for NETosis; tumor itself can also induce NET formation (37). In addition to directly contacting with tumor cells, NETs may be involved in coagulation abnormality of patients with gastric cancer. The NET levels of gastric cancer patients have been found to be significantly correlated with thrombin-antithrombin complex levels and D-dimers, indicating that NETs may play a procoagulant role in the progression of gastric cancer (133). Preoperative blood coagulation status is closely related to lymph node metastasis and disease stage of patients suffering from gastric cancers; this status is also considered to be one of the main causes of death (136). Therefore, targeting NETs therapy may suppress metastasis and recurrence and improve prognosis in gastric cancer patients.

Regarding the relationship between NETs and gastric cancer, existing research shows that NETs contribute to the hypercoagulable state of the circulating system and may promote metastasis of the abdominal cavity and distant organs. However, the direct effect of NETs on gastric cancer cells and tumor angiogenesis and the influence of NETs on other immune cells in the TME remain largely unknown. The related mechanisms of NETs promoting gastric cancer metastasis still require more detailed analysis. A recent study used neutrophils loaded with Abraxane (nanoparticle albumin bound paclitaxel) to serve as a Trojan horse; when they reach the radiated tumor site, the Abraxane could be released into tumor *via* NETosis and exert tumor killing function (137). The above research reveals that researchers should not only explore the role of NETs in tumorigenesis and development but also the utilization of NETs for cancer treatment.

### Colorectal Cancer

Liver metastasis and postoperative recurrence are the main factors for the poor prognosis of colon cancer (138). As mentioned earlier, surgery stress could induce neutrophils to form NETs, which are correlated positively with operative time and intraoperative blood loss (135). Similar to this finding, a study showed that the levels of postoperative circulating NETs are negatively related to the DFS of patients undergoing attempted curative liver resection for metastatic CRC; in addition, abnormal NETs in the tumor are closely correlated with intratumor hypoxia in a murine model (89). Regardless of stimuli, neutrophils from patients with CRC also tend to undergo NETosis *in vitro* compared with those of healthy donors (90). Moreover, poor outcomes of patients are significantly correlated with increased preoperative NET production (103). Those studies revealed that several factors, such as exosome with Kras mutation (59), poly-phosphate (74), and platelet (77), may induce abnormal NET production in the serum of CRC patients, aside from surgery stimulation.

What role does NETs play in the tumorigenesis and development of CRC? Several reports have shown that NETs are involved in CRC progression by promoting tumor cell proliferation, invasion, and adhesion (37, 51, 89, 99). NETs activate the TLR-9 pathway *via* NET-related HMGB1 to promote CRC cell proliferation, adhesion, migration and metastasis, which can be inhibited by DNase I administration (89). Clinical research revealed that NETs are abundant in the liver metastasis of patients with colon cancer, and NET-DNA functions as a pro-tumor factor that promotes tumor cell proliferation, adhesion, migration and metastasis by interacting with the extracellular N terminus of CCDC25 protein in a preclinical murine model (99). NE, one component of NETs, is found to be correlated with CRC progression, and NE concentration is not only significantly higher in the tumor tissues than the normal tissues; but is also higher in the serum of CRC patients compared with those of healthy controls (139). NE has also been found to be a pro-tumor factor and to stimulate the MC38 cell proliferation (103). Furthermore, NETs contribute to the coagulation dysfunction of CRC patients by impairing endothelial cells and activating platelets to shortening coagulation time, and increasing thrombin-antithrombin (TAT) complexes and fibrin fibrils (77). MMP-9, a component of NETs, has been found to promote liver metastasis of colon cancer in an ischemia-reperfusion injury model of the liver, which can be prevented by doxycycline, a broad-spectrum MMP inhibitor (140), further indicating that NETs may be involved in the metastatic process of CRC. On the other hand, NETs are not only capable of modifying the metabolic programming of cancer cells to reduce tumor cell apoptosis and enhance tumor cell growth *in vitro* but can also promote the development of subcutaneous tumor implants and hepatic metastases in mice (103). Although most studies have demonstrated that NETs mainly promote CRC progression, NETs have also been proven to inhibit the proliferation and induce apoptosis of Coca2 cells *in vitro* (85). Although these reports generally indicate that NETs could be involved in CRC progression, such as promoting tumor growth, leading the hypercoagulation state of the circulatory system, and mediating the liver metastasis, the mechanisms by which NETs promote CRC progression are still unclear, and must be confirmed in future research. In the view of NET-associated anti-tumor function, NETs may perform different functions on CRC progression under certain conditions, which must also be further explored.

In addition to studies on the relationship between NETs and CRC, several studies focused on diseases that are closely related to CRC, such as inflammatory bowel disease and intestinal polyps. Chronic inflammation has been proven to promote the malignant transformation of epithelial cells and is closely related to the occurrence of tumors. Crohn’s disease and ulcerative colitis (UC) are the most common inflammatory bowel diseases (IBD), which are closely related to the occurrence of colitis-associated colorectal cancer (141). Considering that IBD is regarded as a risk factor for CRC (142, 143), the role of NETs in the progression of IBD needs to be explored. Studies have shown an abnormal increase of NET-related proteins in fecal samples from IBD patients (144) and in UC colon tissue (145). NET-associated proteins have also been found to be over-expressed in the inflamed colon of UC patients compared with healthy controls. Colitis could be effectively attenuated by NETs inhibition in a dextran sodium sulfate induced mouse model (146). Fecal calprotectin, one component of NET-associated proteins, has been suggested to be a reliable marker for the detection of IBD activity (147). These studies confirm that NETs play a key role in the occurrence and progression of IBD, which is further demonstrated in pediatric IBD (148). However, the specific mechanisms of NETs that promote IBD progression, and whether NETs could accelerate the malignant transformation of IBD require further study. Intestinal polyps and colorectal adenomas, as precancerous lesions of colorectal cancer, have been studied extensively. A study has shown that NETs are involved in the progression of CRC and adenomas with high-grade dysplasia (74). Furthermore, calprotectin has been demonstrated to participate in the progression of colorectal polyps (149). MMP9 has also been found to activate latent TGF-β, which then further suppresses the T-cell activity to promote the occurrence and development of CRC in the model of Apc2^fl/fl-Cdx2CreERT^ mice (150). Prevention is considered one of the best ways to treat cancer. Therefore, further research on the roles of NETs in IBD, polyps, and adenomas are largely helpful to the prevention and treatment of CRC.

In general, NETs have been widely proven to play a critical role in CRC progression, but the specific underlying mechanism remains largely unknown. Meanwhile, NETs have been found to contribute to the development of IBD and polyps which may facilitate tumor formation. Thus, targeting NETs could contribute to the prevention and treatment of CRC. A recent study showed that targeting NET-associated carcinoembryonic Ag cell adhesion molecule 1 (CEACAM1) could inhibit the adhesion, migration and metastasis of colon cancer cells, which indicates that CEACAM 1 may serve as a potential therapeutic target to retard CRC progression (151).

### Liver and Biliary Cancer

Liver cancer is the seventh most frequently diagnosed cancer and the second most common cause of cancer-related death all over the world (14). Among liver cancers, hepatocellular carcinoma (HCC) accounts for 70% to 85% of patients with confirmed liver cancer worldwide. Several factors have been found to be involved in the occurrence of HCC, such as hepatitis B or C virus, metabolic disorders, nonalcoholic steatohepatitis, or alcohol intoxication (152). NETs have been found to contribute to the progression of various chronic liver diseases, such as alcohol-associated liver disease, nonalcoholic fatty liver disease and portal hypertension (153), which are closely related to HCC occurrence, indicating that NETs may play a role in HCC development.

Studies have shown that NETs levels are higher in patients with nonalcoholic steatohepatitis than those in healthy individuals. Analyses on potential mechanism have indicated that early infiltration of neutrophils and the increase of NETs promote the monocyte infiltration and inflammatory cytokines production (82), and induce the differentiation of naïve T cell into Treg by activating TLR4 signaling (154), which then result in malignant transformation of epithelial cells and development of liver cancer. Furthermore, serum NETs in HCC patients are also significantly higher than those in healthy donors (155). Increased NETs have been demonstrated to be closely related to the formation of portal vein thrombosis in patients with HCC and positively correlated with liver disease severity (156). However, a single center, prospective tissue-based study found no correlation between NETs and the venous thrombosis of patients with liver cancer (157). Considering that numerous factors affect coagulation function and that the abnormal liver function itself is also closely related to coagulation abnormality, the relationship between NETs and blood hypercoagulability in patients with liver cancer may require further large-scale clinical studies and more experimental data to determine the effects of NETs on coagulation of live cancer patients. Aside from the capability of NETs to promote malignant transformation of epithelial cells and its involvement in the hypercoagulation status of patients, NETs have also been found to directly stimulate Hepa1-6 and Huh 7 cell proliferation by enhancing mitochondrial function and biogenesis *in vitro*, similar to findings in CRC research (103); NETs could also capture circulating cancer cells, which leads to hepatic malignancy metastasis, depending on the activated platelet (158). Moreover, neutrophils derived from HCC patients, especially advanced HCC, are inclined to release NETs even without any stimulation, and plasma of patients or culture medium of HCC cells could stimulate the release of NETs in neutrophils of healthy donors (38). Another study showed that NETs have no effect on the proliferation of HCC cells *in vitro* but can promote tumor growth *in vivo* by enhancing adhesion, invasion, and angiogenesis of HCC, suggesting that tumor proliferation led by NETs may partly or completely require the participation of other factors in HCC (38). Additionally, cathepsin G associated with NETs improves the invasive ability of HCC by regulating the expression of E-cadherin *in vitro* and *in vivo* (54). These results indicate that NETs are rich in patients with nonalcoholic steatohepatitis and HCC, which play a critical role in the occurrence and development of liver cancer by promoting angiogenesis, adhesion and invasion of the tumor. However, whether NETs promote the hypercoagulable state of the blood is still unclear. Existing studies on the roles of NETs in liver cancer, such as whether NETs are related to liver cancer metastasis and the metabolism of vitamin K, are still not sufficiently comprehensive. Recent studies found that NETs may play a vital role in the formation and development of portal hypertension (PHTN) (159, 160). These findings indicate that whether NETs are involved in the formation of PHTN and liver cancer-associated ascites also needs to be studied. Therefore, in-depth research is still necessary to determine the effect of NETs on liver cancer.

Hepatitis virus infection is widely known as a common cause of liver cancer. Related studies revealed that the peripheral NLR of patients with chronic hepatitis B is positively correlated with disease severity and mortality (161). A recent study showed that HBV infection can inhibit the release of NETs from neutrophils by regulating the levels of cellular ROS and autophagy to maintain chronic liver inflammation (162). The abovementioned studies suggest that neutrophils may affect the outcome of viral hepatitis, but their specific role is still unclear, and whether NETs play a role in the final malignant transformation of viral hepatitis needs to be further explored.

Cholangiocarcinoma and gallbladder cancer also are common malignant tumors of the biliary system. Most patients experience biliary obstruction, worsening of progressive jaundice, and poor prognosis. Research focusing on the role of NETs in those two tumors is limited. Studies indicate that neutrophil infiltration in the tumor is negatively related to the prognosis of patients with cholangiocarcinoma (163, 164). Infiltrative intertumoral neutrophils recruited by CXCL5 could promote tumor metastasis and the recurrence of intrahepatic cholangiocarcinoma (165). Additionally, the level of NETs in the peripheral blood of patients with extrahepatic cholangiocarcinoma is significantly higher than that in healthy people, and NETs levels are positively correlated with the occurrence of thrombosis in patients (166). A recent clinical study showed that the peripheral NLR (NLR≥5) could be regarded as an independent prognostic factor for gallbladder cancer, suggesting poor prognosis (167). Meanwhile, NETs accelerate the formation of thromboembolism in gallbladder cancer (53). Related studies suggest that neutrophils may be involved in the progression of cholangiocarcinoma and gallbladder cancer, but the specific mechanism is still unknown. Whether NETs play a major role in this process requires further study.

### Pancreatic Cancer

Most patients with pancreatic cancer are at an advanced stage at the time of diagnosis, and metastasis and thrombosis are the main factors affecting the prognosis of patients (168, 169). A retrospective cohort study showed that approximately 40% of patients with advanced pancreatic cancer are diagnosed with venous thromboembolism (VTE) after the start of palliative chemotherapy (170). Moreover, VTE may be negatively related to the chemotherapeutic effect, progression-free survival and OS of patients with advanced pancreatic cancer (171). Abnormally increased NETs are observed in pancreatic cancer patients and the models of murine orthotopic pancreatic cancer (95, 172); these NETs can be regarded as an independent prognostic factor for evaluating the outcomes of patients with PDAC, which are negatively correlated with the OS and recurrence-free survival of patients (40). Existing studies showed that increased levels of NETs in pancreatic cancer are partly due to the direct effect of tumor cells, cancer-associated fibroblasts, or activated platelet (67, 76, 79, 106).

NETs have been found to be closely related to blood hypercoagulability in patients with pancreatic cancer (173); and in nude mice with human pancreatic cancer cells (174) by several ways, such as converting endothelial cells toward a procoagulant phenotype (95) and stimulating platelet aggregation (76, 106). Furthermore, NETs have been found to cooperate with procoagulant microparticles released by tumor cells to promote the formation of deep vein thrombosis in murine models of PDAC (175). NET formation could be restricted by inhibiting autophagy with chloroquine or genetic ablation of RAGE, indicating that NETosis in pancreatic cancer may be mainly dependent on autophagy or RAGE expression of neutrophil (172). In addition, a randomized controlled clinical study found that chemotherapy with hydroxychloroquine significantly reduces peri-operative VTE rate (100). These results suggest that NETs could promote the formation of venous thrombosis of pancreatic cancer patients through several ways, which then facilitate disease progression. Therefore, treatment of NETs may reduce the mortality of patients, and improve the quality of life and prognosis of patients with pancreatic cancer.

NETs have been found to significantly promote tumor angiogenesis and potentiate the migration and invasion abilities of AsPC-1 PANC-1 and MMIAPaCa-2 cell *in vitro* (106, 176). NET-associated IL-1β was recently found responsible for the EMT process of pancreatic cancer *via* EGFR-ERK pathway (107). In addition to the direct effect, NETs can facilitate tumor progression by affecting the functions of other cells. NETs could also promote and enable tumor proliferation by stimulating pancreatic stellate cells that form dense fibrous stroma (101). NETs also enhance the migration of hepatic stellate cells, which then promote liver micrometastasis of PDAC (177).

The role of NETs in pancreatic cancer is relatively clear, which is mainly to promote tumor progression. However, the regulation of NETs on tumor cells, angiogenesis, and metastasis remains unclear. Moreover, NETs have been found to impair the sensitivity of PDAC to the treatment of immune checkpoint blockade (PD-1, CTLA4) (72). Thus, whether the combination between targeting NETs and other treatment is beneficial to the prognosis of pancreatic cancer will need to be further explored.

## Discussion

### Insights About This Double-Edged Sword

Like a double-edged sword, NETs have been proven to perform an anti-tumor or pro-tumor function on tumor progression. The determinant of the difference of NETs-associated function on tumor remains unknown. As shown in **Figure 1**, the complexity of inducing factors and components may determine the characteristics of the double-edged sword of NETs in cancer. Therefore, in-depth study on the inducing factors and components of NETs must be conducted, which may gradually reveal the role of NETs in cancer.

Neutrophils are widely known as a type of multi-functional and complex cells; that are not only involved in the innate immune response, but also in modulating the adaptive immune response by directly or indirectly affecting the dendritic cells and lymphocytes (178). Therefore, neutrophils have been considered as a bridge between the innate and adaptive immunity. To determine the role of NETs in cancer, their effect on the innate and adaptive immunity, especially in the tumor immune microenvironment, requires research. NETs have also been found to impair the anti-tumor function of CD8^+^ T lymphocytes and NK cells (47), further indicating that exploring the role of NETs in immune response is crucial for cancer treatment. In addition, whether NETs can affect the function of macrophages and dendritic cells (DCs) needs to be determined. NETs could dampen the LPS-induced maturation of DCs, whereas DCs also could promote the degradation of NETs, suggesting that an interaction transpires between NETs and DCs (179). Furthermore, a study has shown the roles of NETs in macrophages and DCs may be partly dependent on the duration of NETs. When exposed to NETs, macrophages and DCs have been activated at an early time (30min) but induced to apoptosis at a later time (6 and 24 hours) (180). These results indicate that NETs may impair the function of both antigen-presenting cells (APCs) and can further delay their own degradation process. However, studies on the relationship and interaction between NETs and APCs in the context of tumor are limited and may need further exploration.

Notably, neutrophils show distinct phenotypic and functional properties at different times of the day, in a process named as aging (181). Approximately 40% of neutrophils in the blood of healthy individuals express CD177 protein (182). Furthermore, CD177^+^ neutrophils have been found to be prone to NETs formation and to produce higher levels of IL-22 and TGF-β compared with CD177^-^ neutrophils (183). In addition, several subsets of neutrophils have been found in the context of cancer, such as tumor-associated neutrophils (TAN) and myeloid-derived suppressor cells (MDSC) (178, 184). The idea that neutrophils are a homogeneous population can therefore be considered unreliable. Consequently, several questions still exist, such as whether each subset of neutrophils can produce NETs upon stimuli, and whether the produced NETs are different from each other. These questions need to be addressed in future research to clarify the role of NETs in cancer.

Overall, although further research is necessary, NETs have been confirmed to play a role in tumors, in terms of both anti- and pro-tumor functions. Most of the existing studies suggest that NETs mainly play a role in promoting tumors, and targeting NETs may serve as a hopeful treatment for tumors. Therefore, exploring ways to target NETs may be beneficial for cancer treatment.

### Strategies to Suppress NETs Formation

Based on the theory that NETs mainly fuel tumor progression (**Figures 1** and **2**), the role of NETs in tumors can be reversed by interfering with NET formation from the perspective of the mechanism of NET formation or from the tumor-related factors that induce NETosis. Theoretically, five strategies can be adopted to inhibit NET formation.

First, the abnormally increased NETs in the tumor area can be reduced by inhibiting the infiltration of neutrophils into the tumor site. Second, the related factors of tumor that induce NET formation (**Table 1**) can be targeted to reduce the abnormal levels of NETs in the tumor site and circulatory system. Third, NET formation can be interfered by targeting neutrophils themselves, such as inhibiting autophagy with chloroquine (172) or disturbing the function of PAD4 protein by GSK484 (92) or BMS-P5, a novel PAD4 inhibitor (185). Furthermore, the pro-tumor effects of NETs can be suppressed by blocking the signaling pathways (**Figure 1**) by which NETs act as a pro-tumor stimulation. Lastly, accelerating the degradation of NETs can help in attenuating the tumor-promoting effects of NETs, such as DNase I administration (37, 48, 101). Recently, liver metastasis of CRC can be inhibited by gene therapy of adeno-associated virus vector which specifically expresses DNase I in the liver (186), further suggesting that treatment with DNase I is a potential cancer treatment.

Served as an arm of neutrophils, NETs notably play an important role in the infection. Although IP administration with DNase I or PAD4 inhibitor has been demonstrated to be safe in several murine models of cancer, whether systemic administration will increase the infection rate remains unclear. Considering the safety of targeting NETs in clinical applications, the clinical application of existing NETs inhibitors may first be used for the adjuvant treatment of tumors. Dornase alfa, a recombinant desoxyribonuclease, has been approved by the US Food and Drug Administration (FDA) for the treatment of cystic fibrosis, suggesting that administration with DNase I is tolerable and safe for patients. Additionally, several clinical trials are exploring the effectiveness of DNase I nebulized inhalation to reduce lung symptoms in patients with respiratory distress syndrome (NCT03368092) or COVID-19 (NCT04541979, NCT04402970, NCT04387786, NCT04359654). Furthermore, Danirixin (GSK1325756), a selective CXCR2 antagonist, has been found to alleviate the symptoms of patients with chronic obstructive pulmonary disease (COPD) by inhibiting the formation of NETs (NCT03250689) (187). Moreover, sivelestat, a NE inhibitor, which has been used in operative patients to attenuate post-operative complications (NCT01170845), is also promising for cancer treatment.

In summary, exploring the specific role of NETs in tumors is top priority. Meanwhile, it is necessary to study the effectiveness of existing targeting NETs treatment in cancer. Finally, the role of NETs in predicting tumor occurrence and prognosis cannot be ignored.

## Author Contributions

YC and LH collected related papers and wrote the initial manuscript. XQ contributed to the figure and table. GW and JZ designed the study and revised the manuscript. All authors contributed to the article and approved the submitted version.

## Funding

This work was supported by the National Natural Science Foundation of China (81871869 and 82072814); Jiangsu Province Social Development Key Projects (BE2020641 and BE2020640); Key Research Development project of Xuzhou (KC19082); the Natural Science Key Project of Jiangsu Provincial Education Department (19KJA470001); Youth Technology Innovation Team of Xuzhou Medical University (TD202003); Jiangsu Provincial Key Medical Discipline, The Project of Invigorating Health Care through Science, Technology and Education (ZDXKA2016014 and CXTDA2017034); Postgraduate Research & Practice Innovation Program of Jiangsu Province (KYCX20_2458) and the Qing Lan Project of Jiangsu Province.

## Conflict of Interest

The authors declare that the research was conducted in the absence of any commercial or financial relationships that could be construed as a potential conflict of interest.

## Publisher’s Note

All claims expressed in this article are solely those of the authors and do not necessarily represent those of their affiliated organizations, or those of the publisher, the editors and the reviewers. Any product that may be evaluated in this article, or claim that may be made by its manufacturer, is not guaranteed or endorsed by the publisher.
